# STAT1β modulates the tumor immune microenvironment to improve prognosis in ovarian cancer: a comprehensive study of transcriptional and protein expression differences

**DOI:** 10.1186/s13048-025-01780-6

**Published:** 2025-08-23

**Authors:** Ning Lan, Xintong Li, Yifan Qiao, Siyi Zhang, Min Chen, Xiaofeng Yang, Yuliang Zou, Juan Ren, Meili Pei

**Affiliations:** 1 Department of Radiotherapy, The First Affiliated Hospital of Xi’an Jiaotong University, Xi’an, 710061 Shaanxi Province PR China; 2 Department of Gynecology and Obstetrics, The First Affiliated Hospital of Xi’an Jiaotong University, Xi’an, 710061 Shaanxi Province PR China; 3 Department of Medical Oncology, The First Affiliated Hospital of Xi’an Jiaotong University, Xi’an, 710061 Shaanxi Province PR China

**Keywords:** STAT1, Ovarian cancer, Tumor immune microenvironment, Causal mediation analysis

## Abstract

**Objectives:**

As a central regulator of the JAK/STAT signaling pathway, STAT1 generates functionally distinct α and β protein isoforms through alternative splicing. We systematically investigated the expression patterns and prognostic relevance of STAT1 isoform-specific transcripts across pan-cancer tissues, with a particular focus on ovarian cancer (OV), and elucidated their potential mechanisms in modulating the tumor immune microenvironment (TIME) to influence patient prognosis.

**Methods:**

Integrated analysis of the GTEx and TCGA databases was conducted to systematically evaluate the transcript expression profiles of STAT1 isoforms across 32 cancer types and 29 normal tissues. Clinical prognostic significance was analyzed using Cox proportional hazards regression models, complemented by multi-omics approaches including functional enrichment analysis, Tumor immune dysfunction and exclusion (TIDE) immune signature evaluation, and mediation effect models. At the experimental validation level, protein expression differences were assessed by Western blotting, while dynamic changes in immune cell infiltration were examined via immunohistochemistry (IHC) and multiplex immunofluorescence.

**Results:**

In the GTEx/TCGA combined dataset, the transcriptional level of the STAT1α isoform was significantly higher than that of the β isoform in most tissues. However, Western blot revealed elevated expression of the STAT1β isoform in OV tissues and cell lines—a phenomenon that may reflect isoform-specific variations in mRNA translation efficiency and/or protein stability. IHC and multiplex immunofluorescence analyses demonstrated that STAT1 expression promoted CD8^+^ T-cell infiltration, suppressed M2 tumor-associated macrophages (M2-TAMs), and remodeled the immune microenvironment. Multivariate Cox regression analysis identified the β isoform as an independent protective prognostic factor in OV (HR = 0.74, 95%CI: 0.55–0.99, *p* < 0.05), and correlation analysis with TIDE immune scores showed higher absolute correlation coefficients for STAT1β. Mediation analysis indicated that STAT1β improved prognosis by reducing T-cell dysfunction (mediation: 26.53%) and inhibiting M2-TAMs infiltration (mediation: 48.07%). These effects may stem from underlying molecular mechanisms involving modulation of PD-L1/PD-1 signaling and transcriptional interference with CSF1-dependent differentiation signals.

**Conclusion:**

The STAT1β isoform plays a more critical role in OV progression and immunomodulation, with its prognostic protective effect closely linked to the regulation of the immune microenvironment. These findings underscore the functional heterogeneity of STAT1 isoforms and their distinct roles in tumor immunology. Future research should prioritize the development of subtype-specific diagnostic tools and targeted therapeutic strategies to advance the development of precision immunotherapies.

**Supplementary Information:**

The online version contains supplementary material available at 10.1186/s13048-025-01780-6.

## Introduction

Signal transducer and activator of transcription 1 (STAT1), a vital component of the Janus kinase (JAK)/STAT signaling pathway, mediates diverse cellular processes by transmitting biological effects of cytokines including interferons (IFNs) [1–3]. This canonical signaling cascade initiates when ligand-receptor binding triggers autophosphorylation and/or trans-phosphorylation of JAKs (non-receptor tyrosine kinases), subsequently inducing phosphorylation events on intracellular receptor domains and STAT proteins. These sequential modifications drive STAT homo- or hetero-dimer formation, nuclear translocation, and ultimately transcriptional activation of target genes [2, 3]. For example, STAT1 plays a critical role in mediating IFN-induced PD-L1 transcription in cancer cells, cytotoxic T lymphocyte recruitment, T cell activation and tumor immune escape [4–6].

The human *STAT1* gene generates two functionally distinct isoforms through alternative splicing: full-length STAT1α (91 kDa) and C-terminally truncated STAT1β (84 kDa) [7]. The latter lacks 38 amino acids which includes the Ser727 phosphorylation site and much of the transactivation domain (TAD). While tyrosine 701 (Tyr701) phosphorylation is indispensable for STAT1 dimer nuclear translocation, Ser727 phosphorylation within the TAD exhibits dual regulatory effects on transcriptional activity [8]. Contrary to traditional understanding of STAT1β as a transcriptionally inert dominant-negative regulator [9], emerging evidence reveals its autonomous transcriptional capacity. Semper et al. demonstrated that STAT1β can independently activate ~ 50% of IFN-γ-responsive genes (e.g., Irf1, Gbp2), accompanied by prolonged Tyr701 phosphorylation and extended chromatin binding duration [8]. Supporting this paradigm shift, Meissl et al. uncovered isoform-specific functions in natural killer (NK) cell biology using transgenic mouse models, where STAT1α proved indispensable for NK maturation and antitumor activity, while STAT1β exhibited partial functional redundancy at diminished capacity [10].

Zhang et al. systematically evaluated the association between STAT1 expression and survival outcomes in patients with solid tumors by integrating literature meta-analyses with The Cancer Genome Atlas (TCGA) database [11]. Their study demonstrated that high STAT1 expression was significantly associated with poor overall survival (OS) in patients with renal carcinoma, low-grade glioma, and lung adenocarcinoma et al. In contrast, elevated STAT1 levels correlated with prolonged OS in high-grade serous ovarian carcinoma, oral squamous cell carcinoma, and rectal adenocarcinoma et al. Ovarian cancer (OV), the most lethal gynecologic malignancy, presents significant therapeutic challenges due to frequent late-stage diagnosis and high recurrence rates [12]. Despite reports associating high STAT1 expression with improved OS in OV, experimental data showed STAT1 may promote tumorigenesis [13]. We hypothesize that these discrepancies stem from distinct functional roles of STAT1 isoforms.

To test this hypothesis, we aim to elucidate differences in expression profiles of the two protein-coding STAT1 isoforms (α and β) across healthy and tumor tissues. By integrating RNA sequencing datasets from TCGA and the Genotype-Tissue Expression (GTEx) project, we systematically evaluate isoform-specific expression patterns in 32 cancer types and 29 matched normal tissues. Furthermore, we correlate isoform expression levels with clinical parameters in OV patients, including OS, pathological stage, metastatic status, and immune infiltration levels, to unravel the potential mechanistic contributions of each isoform to tumor progression.

## Materials and methods

### Data sources

The transcript expression RNAseq data in the GTEx (https://gtexportal.org/home/) and the TCGA (https://portal.gdc.cancer.gov/) were retrieved from the “TCGA TARGET GTEx” study of UCSC Xena Toil RNA-Seq Recompute Compendium (https://xenabrowser.net/) (accessed on March 20, 2025) [14]. The transcript expression RSEM TPM dataset and OS status and time of patients with cancer were downloaded from UCSC Xena, containing 10,535 samples from TCGA. In addition, the phenotype data of OV were also downloaded from UCSC Xena. After the screen criteria, 352 OV samples were obtained to further analyses. The STAT1 and BRCA1/2 mutation profiles of pan-cancer samples interrogated via the cBioPortal platform (https://www.cbioportal.org/).

This study collected the ovarian tissues of 46 OV patients and 43 benign gynecological diseases who underwent ovariectomy in the First Affiliated Hospital of Xi’an Jiaotong University from 2021 to 2022 (the detailed clinicopathological features of the OV patients are shown in Supplementary Table S1, The cohort contain 76.7% Stage III/IV, 93.0% high-grade serous carcinoma subtype.). This study was approved by the ethics committee of the First Affiliated Hospital of Xi’an Jiaotong University (approval number: 2020-G143). All selected patients signed informed consent, and the study was conducted in accordance with the declaration of Helsinki.

### Cell culture

The human OV cell lines (including A2780, ES2, OVCAR8, OVCAR3, HEY, HO8910, SKOV3, CAOV3) and the normal ovarian epithelial cell line IOSE80 were provided by the Precision Medicine Center at the First Affiliated Hospital of Xi’an Jiaotong University. The cell lines were cultured in Dulbecco’s modified Eagle’s medium (DMEM) or RPMI-1640 medium containing 10% fetal bovine serum (FBS) and 1% penicillin/streptomycin. All of the cell lines were grown in an incubator at 37 °C with 5% CO2.

### Western-blot

Western-blot analysis was conducted by extracting total protein from cells using a Radio Immunoprecipitation Assay (RIPA) lysis buffer (AccuRef Scientific, Xi’an, China), supplemented with protease inhibitors. Following protein quantification via nanodrop, an equal amount of protein was subjected to SDS-PAGE for electrophoretic separation. Subsequently, the resolved protein bands were transferred onto a polyvinylidene fluoride (PVDF) membrane (Millipore, Massachusetts, USA) employing a semi-dry transfer technique. The membrane was then blocked with 5% skimmed milk (Yili Industrial Group Co., Ltd., China) before incubation with primary antibodies, followed by secondary antibodies conjugated to horseradish peroxidase. The visualization of protein bands was achieved using ECL chemiluminescent reagents (Biosharp, Hefei China). The primary antibodies utilized in this experiment included anti-STAT1 (1:1000, Proteintech, Wuhan, China), anti-phospho-STAT1-Y701 (1:1000, ABclonal, Wuhan, China), anti-phospho-STAT1-S727 (1:1000, Abways, Shanghai, China) and anti-GAPDH (1:5000; Servicebio, Wuhan, China). The STAT1 antibody recognizes an immunogen domain spanning amino acids 2-230 and is capable of detecting both α and β isoforms.

### Immunohistochemistry (IHC) and multiplex immunofluorescence staining

For immunohistochemistry (IHC) staining, paraffin-embedded 4-µm-thick sections were deparaffinized, rehydrated in graded alcohol, blocked by endogenous peroxidase blocking solution and subjected to antigen retrieval using 10 mM citrate buffer. TMA slides were then blocked with goat serum for 2 h at 37°C and incubated overnight with primary antibody: STAT1 (1:200, Proteintech, Wuhan, China) and CD8a (1:150, Servicebio, Wuhan, China) at 4°C. Subsequently, slides were incubated with a horseradish peroxidase (HRP)-conjugated secondary antibody, followed by stain using 3,3’-diaminobenzidine solution. Finally, slides were counterstained with hematoxylin and mounted with neutral balsam. The IHC staining score of each sample was calculated as multiplying the staining intensity by the proportion of the positive staining cells [15, 16]. The staining intensity was graded as follows: 0 (negative), 1 (weak), 2 (moderate) and 3 (strong), and the proportion of the positive staining cancer cells was scored as follows: 1 (1–25%), 2 (26–50%), 3 (51–75%) and 4 (76–100%). The scores of each tumor sample were multiplied to give a final score of 0–12 [16], and the tumors were finally determined according to their STAT1 expression as low expression, score < 6, and positive expression, score ≥ 6. Two pathologists, without prior knowledge of the clinical data, independently graded the staining intensity in all cases. For each tumor specimen, four representative 20× microscopic fields were analyzed, systematically covering both the tumor center and invasive front.

Multiplex immunofluorescence staining was performed using Tyramide Signal Amplification (TSA) on formalin-fixed paraffin-embedded tissues. After deparaffinization, antigen retrieval was conducted under microwave heating in EDTA buffer (pH 8.0), followed by endogenous peroxidase blocking with 3% H_2_O_2_. Four iterative staining cycles were performed, each consisting of serum blocking (3% BSA or 10% rabbit serum depending on primary antibody species), overnight primary antibody incubation, HRP-conjugated secondary antibody treatment, TSA-based fluorescent tyramide labeling (utilizing different fluorophores: iF440, iF488, iF555, and iF647; 10 min/dark), and antibody stripping to remove bound antibodies. After the cycles, nuclei were stained with DAPI, autofluorescence was quenched, and slides were mounted for imaging. The slides were imaged using a scanner, and the acquired image data were analyzed using CaseViewer (v2.4, 3DHISTECH) software.

### Survival analysis and cox regression analysis

The “surv_cutpoint” function from the “survminer” R software package was utilized to ascertain the optimal cut-off values for the variables of interest. Based on these values, patients were stratified into high and low expression groups, and a Kaplan-Meier curve was plotted to compare survival rates between the two expression levels. Univariate and multivariate Cox proportional hazards regression models were conducted using the “coxph” function from the “survival” package to assess the potential of different STAT1 transcript expression levels as independent prognostic biomarkers in OV patients. The multivariate analysis incorporated factors such as age, stage, tumor grade, and transcriptional expression levels. Subsequently, a forest plot generated by the “forestplot” R software package clearly depicted the *p*-values, hazard ratios (HRs), and 95% confidence intervals (CIs) for each variable considered.

### Exploration of TIDE signatures

By utilizing the Tumor Immune Dysfunction and Exclusion (TIDE, http://tide.dfci.harvard.edu/) database [17], we evaluated the TIDE score, dysfunctional cytotoxic T cells score, exclusion cytotoxic T cells score, interferon gamma (IFNG) score, M2 subtype of tumor-associated macrophages (TAMs) score, cancer-associated fibroblasts (CAFs) score, and myeloid-derived suppressor cell (MDSC) infiltrations score, CD274 (PD-L1), CD8, and microsatellite instability (MSI) score. These indicators collectively reflect the anti-tumor and tumor immune escape capabilities of each OV sample.

### Correlation analysis and functional enrichment analysis

Spearman correlation analysis was used to determine the correlation between the expression levels of different transcripts and various immune prediction scores of TIDE. The top 100 genes most significantly correlated with STAT1 (ranked by descending order of absolute Pearson’s correlation coefficient, requiring *p* < 0.05 and |r| >0.55) were selected for functional enrichment analysis. The “clusteprofiler” package in R was used to check the cell component (CC), molecular function (MF) and biological process (BP) categories in the gene ontology (go). The “simplification” function in the R package is used to reduce redundancy in the output of rich go terms.

### Statistical tests

Data analysis in the study was conducted primarily using R language (version 4.2.2). The “ggplot2” package facilitated figure creation. The Wilcoxon test estimated statistical significance for quantitative data comparisons between two types. Kaplan-Meier survival analysis employed the log-rank method to determine statistical significance. Values of *p* < 0.05 were deemed statistically significant, with significance levels denoted as *p < 0.05, **p < 0.01, ***p < 0.001, and ****p < 0.0001.

To investigate whether various immune scores could serve as mediators explaining the relationship between independent variables (transcript expression levels of STAT1 α/β subtypes) and dependent variables (patient survival outcomes), we conducted causal mediation analysis using the “mediation” package in R. Specifically, under the counterfactual framework of causal mediation analysis, the total effect of exposure variables (different transcript expression levels) on the outcome variable (overall survival rate, OS) was decomposed into direct effects and indirect effects mediated through different scores. All mediation analyses employed a quasi-Bayesian Monte Carlo method with 1,000 simulations based on normal approximations. This method estimates uncertainty in mediation effects through repeated simulations, accounting for potential confounding. Results present the effect magnitude and *p*-values for average direct effects (ADE) and average causal mediation effects (ACME), along with the proportion of mediated effects.

## Results

### Expression profiling of STAT1 splice variants across human tissues and cancers

**Fig. 1:**
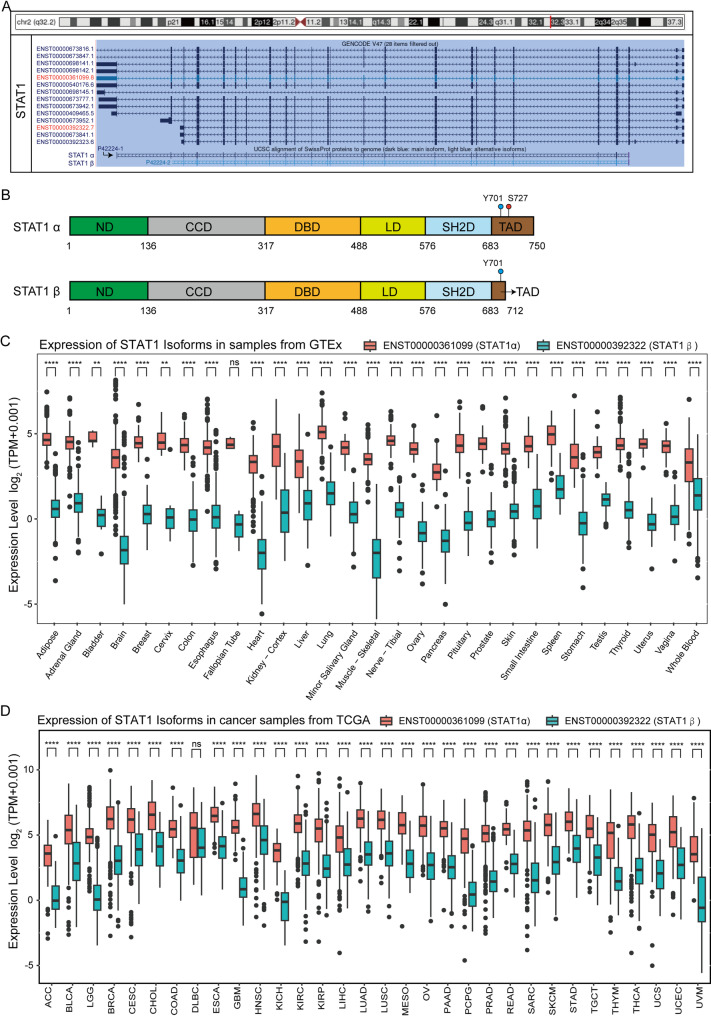
STAT1 classical transcripts and expression analysis in different tissues. (**A**) Fourteen gene transcripts of *STAT1*, as cataloged by UCSC, were identified. (**B**) Schematic representations of two classic *STAT1* transcripts and their protein isoforms domains were presented. (**C-D**) Boxplots depict STAT1α/β transcript expressions (log_2_TPM) in normal (**C**: *n* = 29 tissues) and tumor (**D**: *n* = 32 cancers). Stats: Wilcoxon rank-sum test. **p < 0.01, ****p < 0.0001, ns: non-significant

Human STAT1 generates at least 14 mRNA transcripts through alternative splicing (Fig. 1A). Among these, 12 isoforms were quantitatively detected in the TCGA-TARGET-GTEx merged RNA sequencing dataset using the UCSC Xena platform (Toil RNA-Seq recompute pipeline). Distinct STAT1 splicing variants typically encode proteins with divergent domain architectures that influence their biological functions. Stacked plot analysis (Supplementary Fig. 1A, B) revealed ENST00000361099 (encoding the α isoform) and ENST00000392322 (encoding the β isoform) as dominant constituents across most samples, with minor isoforms (cumulative expression < 5%) grouped as “others”. Structurally, the α isoform (UniProt ID: P42224-1) comprises 750 amino acids (91 kDa), whereas the β isoform (UniProt ID: P42224-2) lacks residues 713–750 (C-terminal truncation removing the Ser727 phosphorylation site and majority of the transactivation domain [TAD]), resulting in a 712-amino acid polypeptide (84 kDa) (Fig. 1B). Notably, transcriptional quantification across 32 TCGA tumor types (excluding DLBC) and GTEx normal tissues demonstrated significantly higher α isoform expression compared to β (*p* < 0.001; Fig. 1C, D), suggesting that the α isoform likely plays the predominant functional role under basal physiological conditions.

### Tumor-specific upregulation and prognostic significance of STAT1 isoforms in OV

**Fig. 2:**
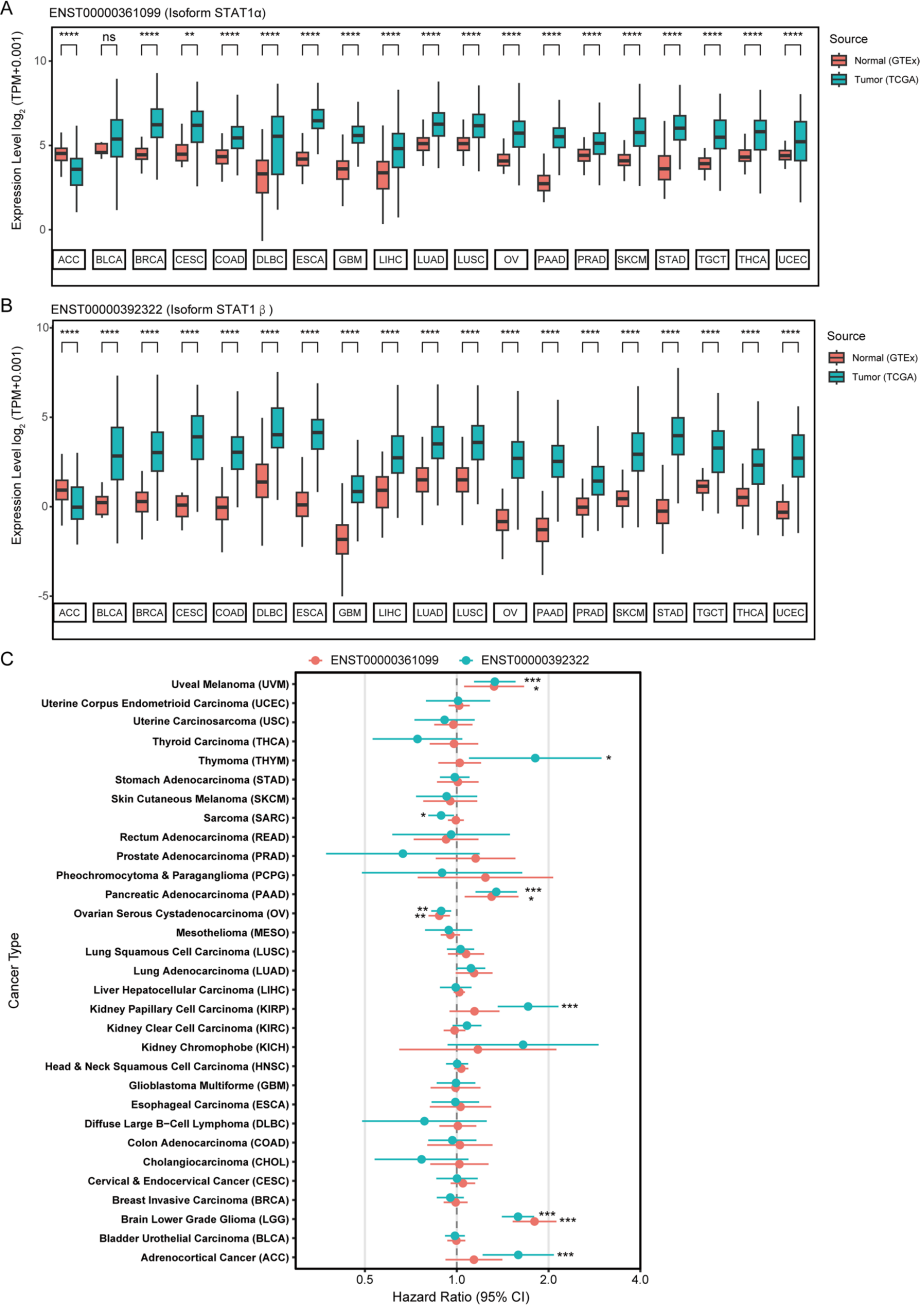
Expression analysis and prognostic significance of *STAT1* classical transcripts across pan-cancer. (**A-B**) Comparison of ENST00000361099 (encoding the isoform STAT1α) (**A**) and ENST00000392322 (encoding the isoform STAT1β) (**B**) expressions between individual normal tissues and corresponding cancer types. Stats: Wilcoxon rank-sum test. (**C**) Univariate COX regression analysis was conducted considering the expression levels of two classical *STAT1* transcripts. Significance levels denoted as *p < 0.05, **p < 0.01, ***p < 0.001, and ****p < 0.0001

Our matched tumor-normal analysis across 19 tissue pairs demonstrated that adrenocortical carcinoma (ACC) exhibited significantly reduced STAT1 dual-isoform expression compared to normal adrenal tissue (*p* < 0.05). Conversely, 18 malignancies – including OV, breast invasive carcinoma (BRCA), and colon adenocarcinoma (COAD) – displayed pronounced upregulation of α/β isoform transcript levels (Figs. 2A, B). Tissue-specific prognostic discordance was observed via univariate Cox proportional hazards regression: elevated STAT1 isoform expression correlated with adverse outcomes (hazard ratio [HR] > 1) in uveal melanoma (UVM), thymoma (THYM), and pancreatic adenocarcinoma (PAAD), while demonstrating protective associations (HR < 1) in sarcoma and OV (Fig. 2C).

**Fig. 3:**
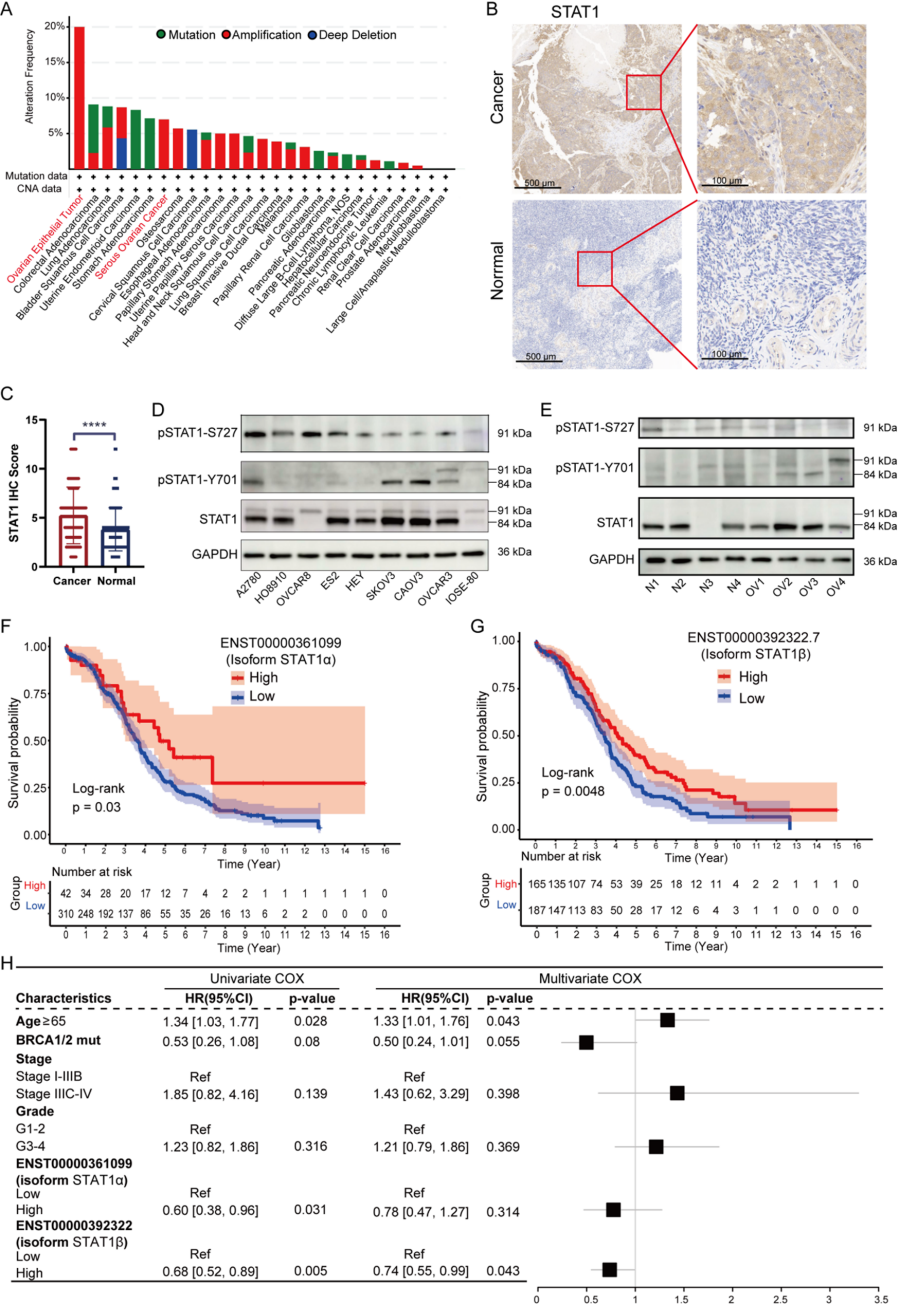
Expression analysis and prognostic significance of *STAT1* classical transcripts for OV patients. (**A**) Genomic alterations of *STAT1* across various tumor types were examined using the “TCGA PanCancer Alters Studies” in the cBioportal database. (**B**) Representative images of STAT1 protein expression in paraffin-embedded tissue from 46 OV samples and 43 normal samples. (**C**) The IHC score of the OV samples and normal ovarian samples. Stats: unpaired two-tailed t-tests, ****p < 0.0001. (**D-E**) Detection of STAT1, phosphorylated STAT1-Y701 (p-STAT1-Y701), and phosphorylated STAT1-S727 (p-STAT1-S727) of different OV cell lines (**D**) and OV tissues or normal ovarian tissues (**E**) through Western-blot analysis. (**F-G**) Kaplan-Meier curves comparing OS between high/low STAT1 isoform groups (stratified by maximally selected log-rank statistic). Stats: Two-sided log-rank test, *p* < 0.05; n (α-low/high) = 310/42, n (β-low/high) = 187/165. (**H**) Multivariate COX regression analysis and forest plots incorporated clinical features and expression levels of these transcripts

cBioPortal genomic profiling identified STAT1 amplifications in 20% of OV cases (Fig. 3A), with amplification-positive tumors showing 1.12-fold (α isoform) and 1.34-fold (β isoform) mRNA elevation (Supplementary Fig. 2A). Clinical validation using tissue samples revealed significantly higher immunohistochemical (IHC) scores in 46 OV tumors versus 43 normal ovarian controls (Figs. 3B, C). Western blot analyses confirmed β isoform overexpression in OV cell lines (SKOV3, OVCAR3) and primary tumors, displaying a significantly greater tumor-to-normal expression ratio than the α isoform (Figs. 3D, E). These data reveal a distinct decoupling between transcriptional and protein expression levels. Post-transcriptional differences in mRNA translation efficiency and/or protein stability underlie the elevated STAT1β protein in OV tissue. The phosphorylation antibody incubation and quantification showed that the phosphorylation level of Y701 site of β subtype in tumor tissues was higher than that in normal tissues, while the phosphorylation level of α subtype with S727 site in fresh cancer tissues was not significantly higher than that in normal tissues (Figs. 3E, Supplementary Fig. 2B). It is noteworthy that the utilized STAT1 antibody (Target: aa 2-230) recognizes an epitope common to both α and β isoforms, thereby reflecting total STAT1 protein levels in IHC analyses. The Western blot data in Figs. 3E demonstrate β-isoform predominance in tumor tissues, suggesting that the observed IHC signals largely represent tumor-associated STAT1β expression.

To assess the impact of expression levels of different variants on patient prognosis, patients were stratified into high- and low-expression groups using the optimal cutoff (Supplementary Fig. 2C, D). Survival analyses indicated that high expression of either isoform correlated with prolonged OS in OV patients (*p* < 0.05, Figs. 3F, G). However, multivariate Cox regression adjusted for clinicopathological variables demonstrated independent prognostic significance only for the β isoform (HR = 0.74, 95% confidence interval [CI]:0.55–0.99, *p* = 0.042), with no statistical relevance for the α isoform (HR = 0.80, 95% CI:0.49–1.32, *p* = 0.388; Fig. 3H). These integrated findings position the β isoform as a key modulator in OV progression.

### Divergent roles of STAT1 isoforms in reshaping the immunological landscape of OV

**Fig. 4:**
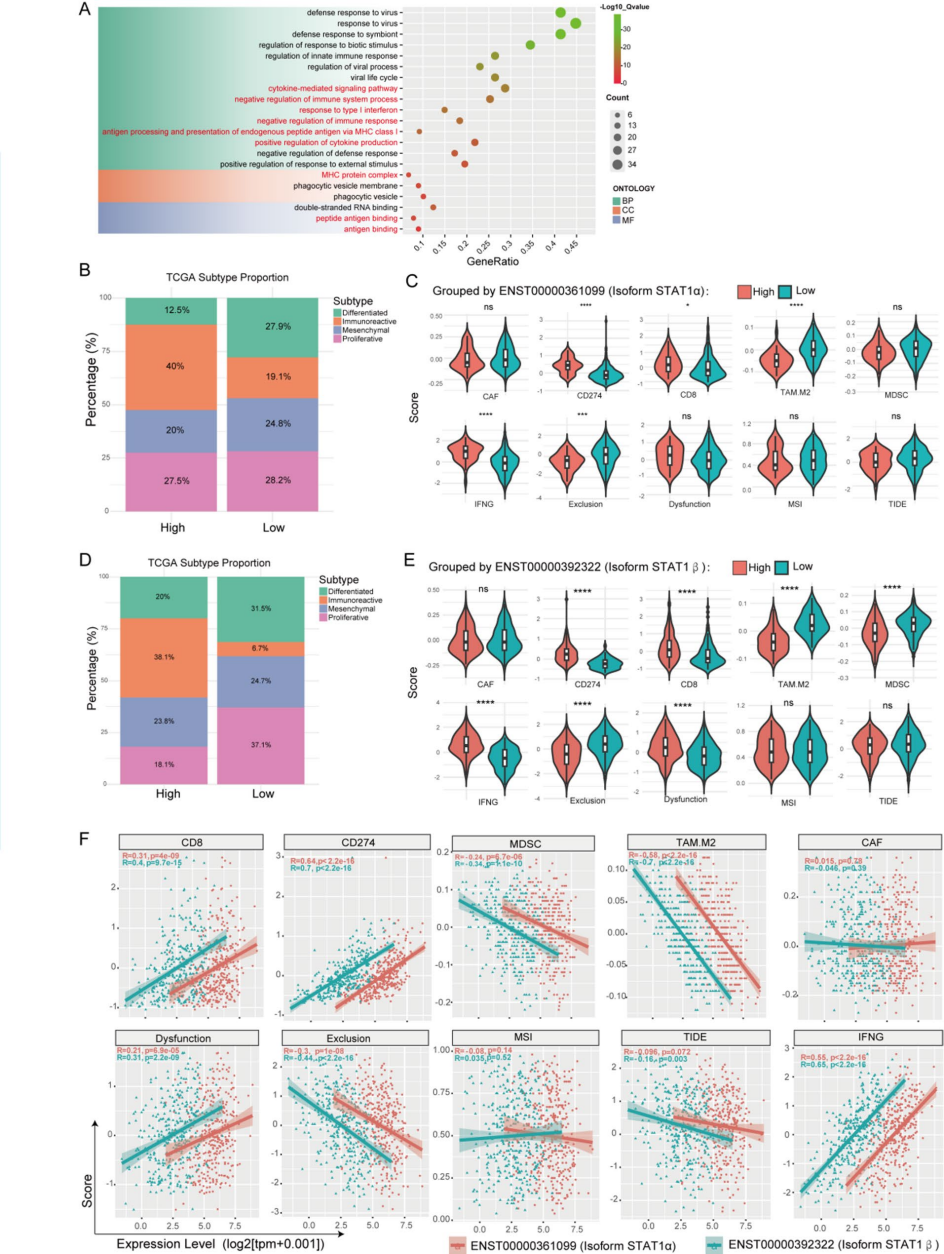
Classical STAT1 Transcripts Correlation with TIME in OV. (**A**) Enrichment analyses of the GO terms for the 100 target genes were conducted. (**B**) Proportions of Murakami molecular subtypes in OV patients stratified by ENST00000361099 expression levels. (**C**) Violin plots compared the signatures of immune microenvironment from TIDE algorithm grouped by ENST00000361099 (encoding the isoform STAT1α). Stats: Wilcoxon rank-sum test. (**D**) Proportions of Murakami molecular subtypes in OV patients stratified by ENST00000392322 expression levels. (**E**) Violin plots compared the signatures of immune microenvironment from TIDE algorithm grouped by ENST00000392322 (encoding the isoform STAT1β). Stats: Wilcoxon rank-sum test. (**F**) Spearman correlation between isoform STAT1α (orange red) expression or isoform STAT1β (green) expression and proportion of CD8^+^T cells, CD274, MDSC, M2-TAMs, CAFs, T cell dysfunction, T cell exclusion, MSI, TIDE, and IFN-γ. *p < 0.05, ***p < 0.001, ****p < 0.0001, and ns: non-significant

To elucidate STAT1’s mechanistic role in OV prognosis, we identified the top 100 co-expressed genes (Pearson’s |r| >0.55) showing strongest associations with STAT1 expression in TCGA high-grade serous ovarian carcinoma cohorts. Gene Ontology (GO) enrichment analysis revealed significant overrepresentation in response to type I interferon (GO: 0034340), antigen binding (GO: 0003823), and MHC protein complex (GO: 0042611) (Q value < 0.001; Fig. 4A), suggesting STAT1-mediated regulation of adaptive antitumor immunity. Using the Murakami molecular classification system [18], both STAT1 isoform-high subgroups demonstrated increased immunoreactive subtype prevalence (α: 40% vs. 19.1%; β: 38.1% vs. 6.7%), with β isoform exhibiting greater discriminative capacity (Figs. 4B, D). TIDE algorithm deconvolution of immune microenvironment features showed that high α/β isoform expression correlated with: Enhanced CD8^+^ T cell infiltration (*p* < 0.05), elevated IFN-γ response signature (*p* < 0.001), upregulated PD-L1 (CD274) expression (*p* < 0.001), reduced M2 TAMs (*p* < 0.001), lower T cell exclusion scores (*p* < 0.001; Figs. 4C, E). As visualized in Fig. 4F, both isoforms exhibited significant correlations with key immune markers, with STAT1β consistently showing stronger effects. To validate these transcriptome-based findings at the tissue level, we assessed STAT1 and immune cell markers via IHC and multiplex immunofluorescence.

**Fig. 5:**
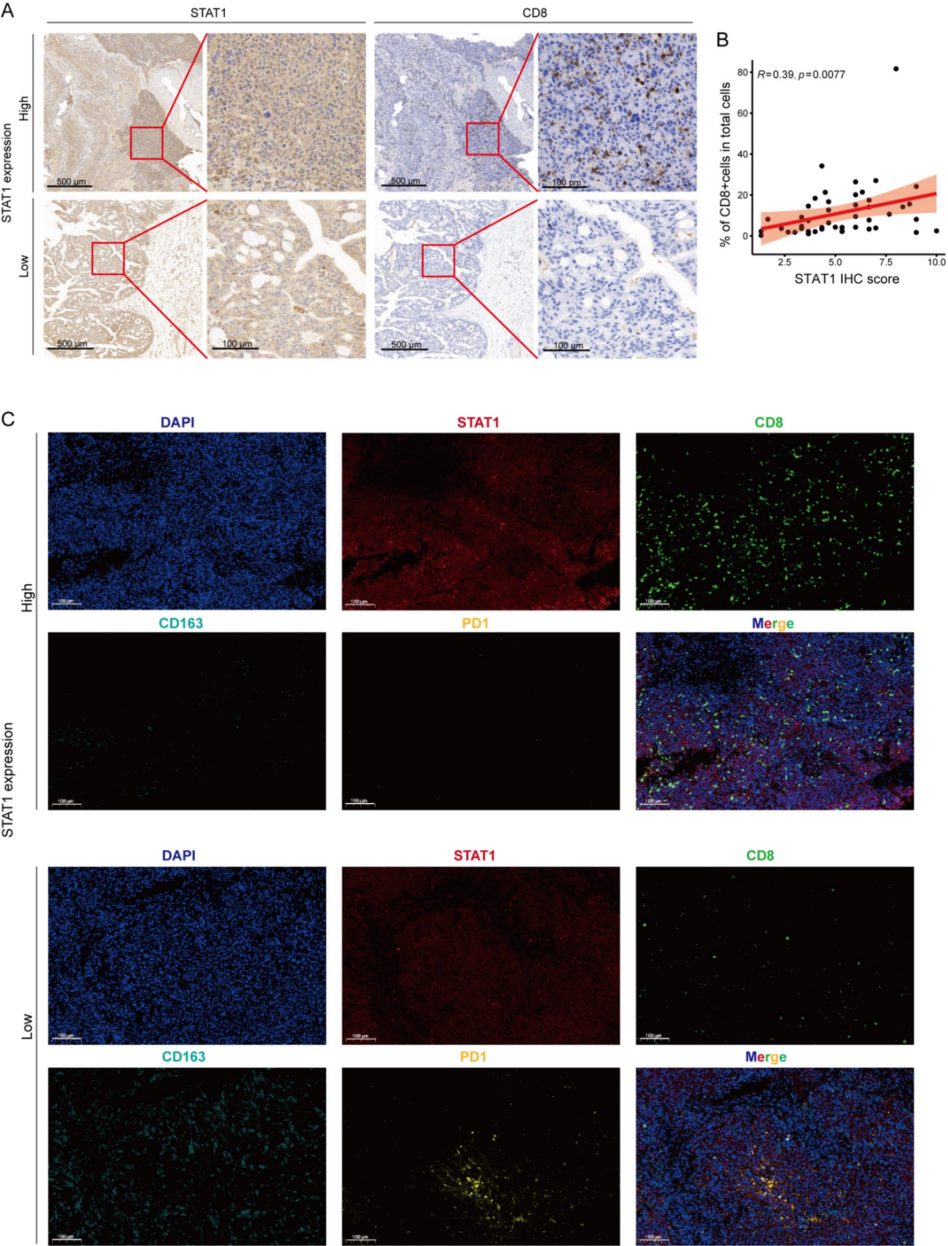
Validation of STAT1 expression correlations with diverse immune markers in patient samples. (**A**) IHC staining for STAT1 and CD8 in OV tissues (46 cases). (**B**) Spearman’s rank correlation analysis of STAT1 IHC scores and CD8^+^ cell proportion. *P* values and R were calculated by Spearman’s correlation analysis. (**C**) Multiplex immunofluorescence analysis of an OV specimen demonstrating spatial colocalization of CD8^+^ T cells (green), CD163^+^ tumor-associated macrophages (cyan), and PD1^+^ lymphocytes (yellow) across differential STAT1 expression gradients (red)

Tissue-level validation via IHC confirmed positive association between total STAT1 protein (pooled α/β isoforms) and CD8^+^ T cell density (*r* = 0.39, *p* = 0.0077; Figs. 5A, B). Given the antibody’s inability to distinguish isoforms, this correlation likely reflects β isoform dominance in tumor cells (Fig. 3E). The Multiplex immunofluorescence of patient tissues further revealed a significant increase in CD8^+^ cells within the high STAT1 regions, accompanied by a decrease in the infiltration of CD163^+^ M2-TAMs and PD1^+^ cells, which were notably enriched in the low STAT1 regions (Fig. 5C). These findings suggest that STAT1 may enhance the microenvironment by promoting the infiltration of CD8^+^ cells and inhibiting immunosuppressive cells such as M2-TAMs, as well as addressing immune cell dysfunction. Given the antibody’s cross-reactivity with α/β isoforms, spatial colocalization patterns (Fig. 5C) should be interpreted as associated with aggregate STAT1 levels. However, the consistent β-isoform transcriptional dominance (Fig. 3D) and its stronger immune correlates (Fig. 4F) support a predominant role of STAT1β in shaping this microenvironment.

### Mechanistic dissection of STAT1 isoform-specific immune regulation

**Fig. 6:**
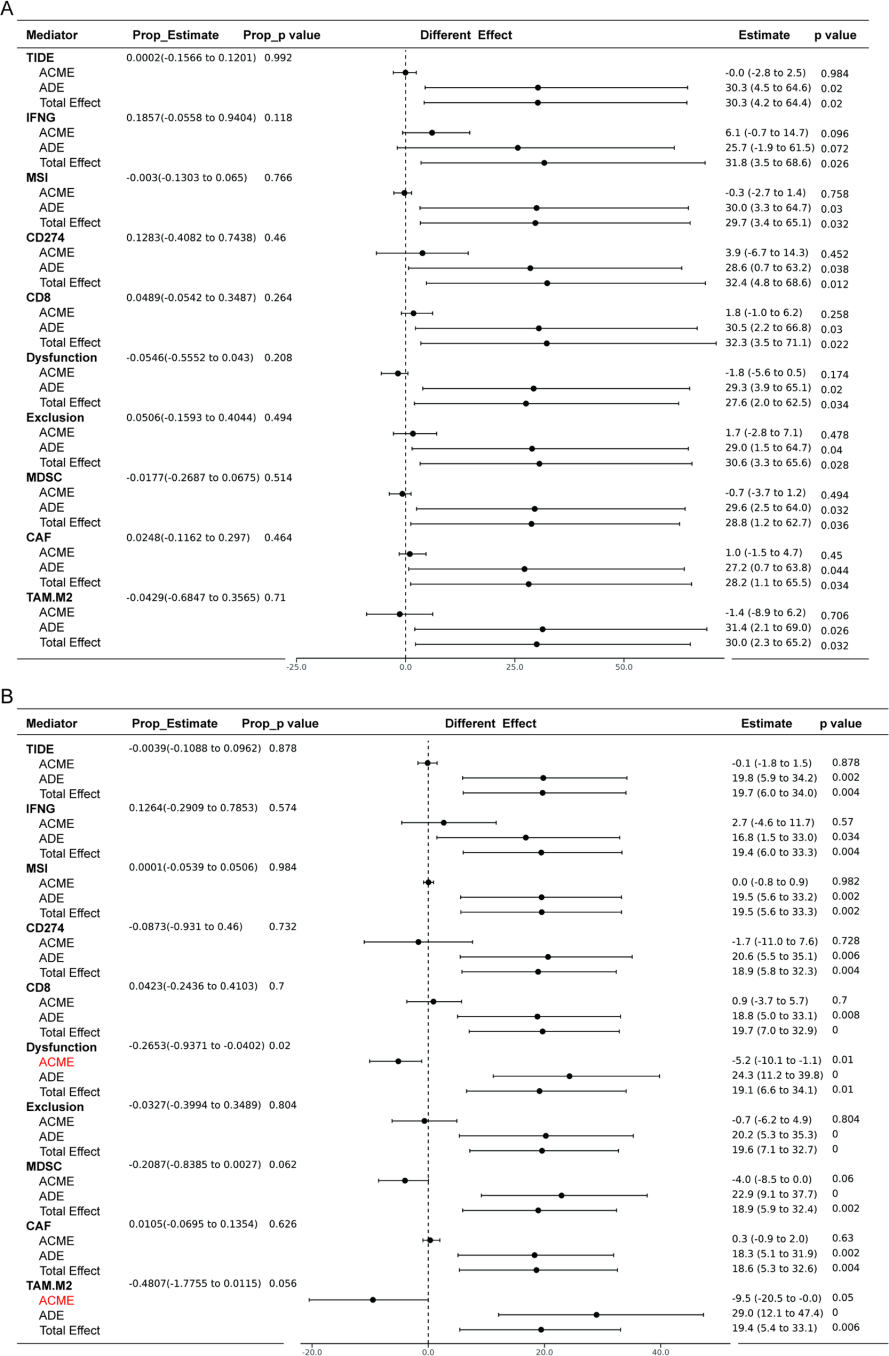
Mediation analyses of the signatures of immune microenvironment from TIDE algorithm between different expression of variants and overall survival of OV patients. (**A-B**) Mediation analysis results based on the expression of the ENST00000361099 (**A**) and ENST00000392322 (**B**). ADE, average direct effect (effect of the expression of different variants on survival not through the TIDE signature); ACME, the average causal mediation effect (effect of the expression of different variants on survival mediated by the TIDE signature)

The limited availability of isoform-specific protein detection tools constrained mechanistic resolution; nevertheless, we performed transcriptome-based causal mediation analysis (R package “mediation”) to quantify immune microenvironment features as potential mediators between STAT1 isoform expression (α/β transcript levels, log_2_TPM) and survival outcomes (Cox proportional hazards model). Bootstrapped results (*n* = 10,000 resamples) revealed differential mediation patterns (Fig. 6A-B): While α isoform’s survival association primarily arose from direct effects (Total Effect = 27.6 ∼32.4), β isoform exhibited significant mediatory contributions through two immune pathways: (1) Mitigation of T cell dysfunction (mediated proportion = 26.53%, ACME=-5.2, *p* = 0.008), consistent with STAT1’s potential modulation of PD-L1/PD-1-mediated T cell activation/exhaustion equilibrium [19–21], and (2) Suppression of M2-TAMs infiltration (ACME=-9.5, *p* = 0.05), likely via transcriptional interference with CSF1 signaling [22]. Notwithstanding, dominant direct effects persisted in both models, suggesting unmeasured tumor-intrinsic mechanisms (e.g., metabolic reprogramming; homologous recombination competence assessed via RAD51 foci formation) potentially underlie isoform-specific prognostic impacts [23, 24]. However, we must acknowledge that causal inference is limited by observational data and may not reflect direct molecular causation without in vivo functional studies. These findings necessitate development of β isoform-selective detection tools (e.g., C-terminal truncation-specific nanobodies) and CRISPR/Cas9-mediated isoform-switching models to dissect non-canonical regulatory functions. In summary, while the inherent integration of signals from both STAT1 isoforms in IHC/mIHC analyses constrains direct mechanistic mapping to individual isoforms, transcript-specific correlations and transcriptome-based mediation provide substantial orthogonal evidence supporting the functional primacy of STAT1β.

## Discussion

In this study, we present the expression profiles of canonical STAT1 isoforms across multiple cancer types and their corresponding normal tissues, and systematically investigate their differential prognostic implications and immunomodulatory mechanisms in OV. In contrast to malignancies such as pancreatic adenocarcinoma (PAAD) and low-grade glioma (LGG), elevated STAT1 expression in OV patients demonstrated significant association with survival advantage (HR = 0.74, *p* < 0.05), with the β isoform transcript exhibiting superior predictive efficacy. Through integrated correlation analysis and mediation effect modeling, we further elucidate that the β isoform potentially mediates its protective prognostic effects via coordinated modulation of CD8^+^ T cell effector functions and M2 TAMs polarization dynamics.

The functional heterogeneity of STAT1 isoforms likely originates from their structural divergence. The β isoform lacks 38 amino acids in the C-terminal transactivation domain (TAD), including the Serine-727 (S727) phosphorylation motif, resulting in distinct signaling kinetics compared to the α isoform. Building on evidence from Zakharova et al., who demonstrated transcriptional activation mediated by recruitment of mediator complexes in a TAD-independent manner for both STAT1α and STAT1β, ChIP assays revealed an exclusive ability of STAT1α to recruit p300 and exhibit full transcriptional activation [25]. These findings collectively indicate a two-step transcriptional activation mechanism coordinated by distinct domains: an initial, STAT1α-specific, TAD-dependent step involving p300-mediated chromatin remodeling, followed by a TAD-independent step common to both isoforms involving the recruitment of transcriptional mediator(s) to initiate transcription. Semper et al. established murine models with isoform-specific expression (STAT1α-only vs. STAT1β-only), demonstrating that STAT1β functions not as a dominant-negative regulator, but as an immunomodulatory molecule with unique transcriptional activity. Comparative analysis showed: Partial functional overlap with STAT1α but differential activation efficiency; 16.7% of IFN-γ-responsive genes (e.g., Plac8 and Gpr33) exhibit strict STAT1α dependence; STAT1β maintains sustained expression of select genes (e.g., Isg20) through prolonged signal persistence. Biochemical characterization revealed enhanced Tyr701 phosphorylation maintenance (up to 32 h) and prolonged DNA binding capacity in STAT1β, contrasting with STAT1α’s rapid deactivation (within 4 h) [8]. This distinctive activation profile may partially underlie the superior prognostic significance of STAT1β in OV progression.

Previous research on the role of STAT1 subtypes in cancer has been relatively limited. A cohort study of OV patients from Brazil indicated that high STAT1 expression in tumors was significantly associated with improved disease-free survival (*P* = 0.0256) and overall survival (*P* = 0.0193), yet this study did not distinguish between the expressions of specific subtypes [26]. Zhang et al. found in esophageal squamous cell carcinoma that STAT1β was positively correlated with total STAT1 expression, and that the STAT1β/α heterodimer could block the proteasome-mediated degradation of STAT1α, thereby amplifying the classical signaling pathway induced by IFN-γ [27]. However, their study did not delve deeply into the impact of subtypes on the tumor microenvironment (TME). In contrast, our study focuses on the field of ovarian tumor immunity and has discovered that the highly selective expression of STAT1β (at the intracellular protein level) and the abundance of its transcripts are significantly correlated with the patient’s immunoreactive phenotype. This aligns with some of the findings of Tian et al., who also verified that STAT1 expression levels in OV cells (such as OVCAR-3 and SK-OV-3) are higher than those in non-cancerous ovarian epithelial cells (HOSEpiC). Furthermore, They confirmed that both STAT1α and STAT1β physically bind to TGF-β receptors (ALK1, ALK5, and TβRII), and that TGF-β1 can dynamically regulate this interaction, enhancing the binding between STAT1β and ALK1 [13]. This may partly explain the more pronounced regulatory role of STAT1β in tumor immunity.

At the immune microenvironment level, our TIDE analysis demonstrated that high expression of the STAT1β isoform is significantly associated with increased infiltration of CD8^+^ T cells, while showing a negative correlation with the abundance of M2-like tumor-associated macrophages (M2-TAMs) and myeloid-derived suppressor cells (MDSCs). This association highlights a potential role for STAT1β in shaping a more immunologically active tumor microenvironment. Previously, STAT1 signaling deficiency (representing overall STAT1 function) has been linked to impaired CD8^+^ T cell recruitment, as seen in LGG where it inhibits the CXCL9/10 pathway [28]. Furthermore, STAT1 deficiency in HNSCC models (Stat1^−/−^ mice) resulted in increased infiltration of MDSCs (CD11b^ +^ Ly6G^+^) and M2 macrophages (F4/80^ +^ CD206^+^), underscoring the importance of STAT1 in restraining these immunosuppressive myeloid populations [20].

The mechanistic significance of STAT1β is further revealed by its strong positive correlation with markers of IFN-γ signaling and PD-L1 expression within the ovarian cancer milieu, as ascertained by our TIDE analysis. This specific correlation suggests a pivotal role for STAT1β in potentiating the IFN-γ/STAT1-IRF1 signaling axis. Enhanced signaling through this axis is crucial for the induction of PD-L1 expression, providing a potential mechanism for our observed link between STAT1β and PD-L1 [21, 22, 29]. Moreover, the negative correlation with M2-TAMs and MDSCs may be functionally linked to STAT1β’s potential to suppress the CSF1 signaling pathway [30, 31]. Based on this evidence, we propose that STAT1β may contribute to an immuno-modulatory microenvironment by (1) fostering PD-L1 expression via the IFN-γ pathway and (2) inhibiting M2 macrophage polarization and MDSC recruitment through antagonism of CSF1 signaling. Altogether, our findings highlight STAT1β as a prognostically favorable, immune-modulating isoform that may represent a viable target for isoform-specific immunotherapy in ovarian cancer.

This study still has the following limitations: (1) The inability of current antibodies to distinguish STAT1α from STAT1β at the protein level (IHC/mIHC) precludes definitive attribution of immune correlations observed in Figs. 3B and 5A and C to STAT1β alone. While transcriptional dominance of STAT1β (Fig. 3D-H) and stronger transcript-level immune correlations (Fig. 4F) support its primacy, this remains an inferential association. Future development of C-terminal truncation-specific antibodies or nanobodies would resolve this constraint. (2) Our mediation models (Fig. 6A-B) implicate STAT1β-specific immune modulation, but these transcriptomic inferences await in vivo validation. CRISPR/Cas9-mediated isoform-switching models (e.g., STAT1β-only OV cells in immunocompetent mice) are essential to resolve whether STAT1β directly orchestrates CD8^ +^ T-cell recruitment and M2-TAMs suppression, or merely correlates with an underlying immunogenic context. (3) Our findings nominate STAT1β as a therapeutic target in OV. Development of isoform-specific agonists should prioritize molecules mimicking STAT1β’s sustained activation kinetics without p300-dependent TAD remodeling.

## Supplementary Information

Below is the link to the electronic supplementary material.

Supplementary Material 1

Supplementary Material 2

## Data Availability

No datasets were generated or analysed during the current study.
