# Supplementary material for: STAT1β modulates the tumor immune microenvironment to improve prognosis in ovarian cancer: a comprehensive study of transcriptional and protein expression differences
Source: J Ovarian Res. 2025 Aug 23;18:192. doi: 10.1186/s13048-025-01780-6 (PMC12374463; doi:10.1186/s13048-025-01780-6)
Supplement: Supplementary file 1 — Supplementary Material 1 [file 13048_2025_1780_MOESM1_ESM.docx]

**Supplemental Table 1.** The clinicopathological features of ovarian cancer patients.

|  | **High group**  **N=19** | **Low group**  **N=24** | **P value** |
| --- | --- | --- | --- |
| **Age** ≥ 55 years | 11 (57.8) | 8 (33.3) | 0.107 |
| **FIGO Stage**  I  II  III  IV | 2 (10.5)  1 (5.2)  13 (68.4)  3 (15.7) | 3 (12.5)  4 (16.6)  15 (62.5)  2 (8.3) | 0.618 |
| **Histology**  Serous  Mucinous  Clear cell  Endometrioid | 18 (94.7)  1 (5.2)  0  0 | 22 (91.6)  0  1 (4.1)  1 (4.1) | 0.414 |
| **BRCA1/2 mut**  Positive  Negative  Unknown | 0  2 (10.5)  17 (89.4) | 1 (4.1)  3 (12.5)  20 (83.3) | 0.646 |
