# Supplementary material for: STAT1β modulates the tumor immune microenvironment to improve prognosis in ovarian cancer: a comprehensive study of transcriptional and protein expression differences
Source: J Ovarian Res. 2025 Aug 23;18:192. doi: 10.1186/s13048-025-01780-6 (PMC12374463; doi:10.1186/s13048-025-01780-6)
Supplement: Supplementary file 2 — Supplementary Material 2 [file 13048_2025_1780_MOESM2_ESM.docx]

**STAT1β Modulates the Tumor Immune Microenvironment to Improve Prognosis in Ovarian Cancer: A Comprehensive Study of Transcriptional and Protein Expression Differences**

Ning Lan^1#^, Xintong Li^2#^, Yifan Qiao^3^, Siyi Zhang^2^, Min Chen^1^, Xiaofeng Yang^2^, Yuliang Zou^2^, Juan Ren^1^, Meili Pei^2*^

^1^Department of Radiotherapy, The First Affiliated Hospital of Xi’an Jiaotong University, Xi’an, Shaanxi Province 710061, PR China

^2^Department of Gynecology and Obstetrics, The First Affiliated Hospital of Xi’an Jiaotong University, Xi’an, Shaanxi Province 710061, PR China

^3^Department of Medical Oncology, The First Affiliated Hospital of Xi’an Jiaotong University, Xi’an, Shaanxi Province 710061, PR China

*Correspondence: Meili Pei, peiml@xjtufh.edu.cn

**^#^ Contributed equally.**


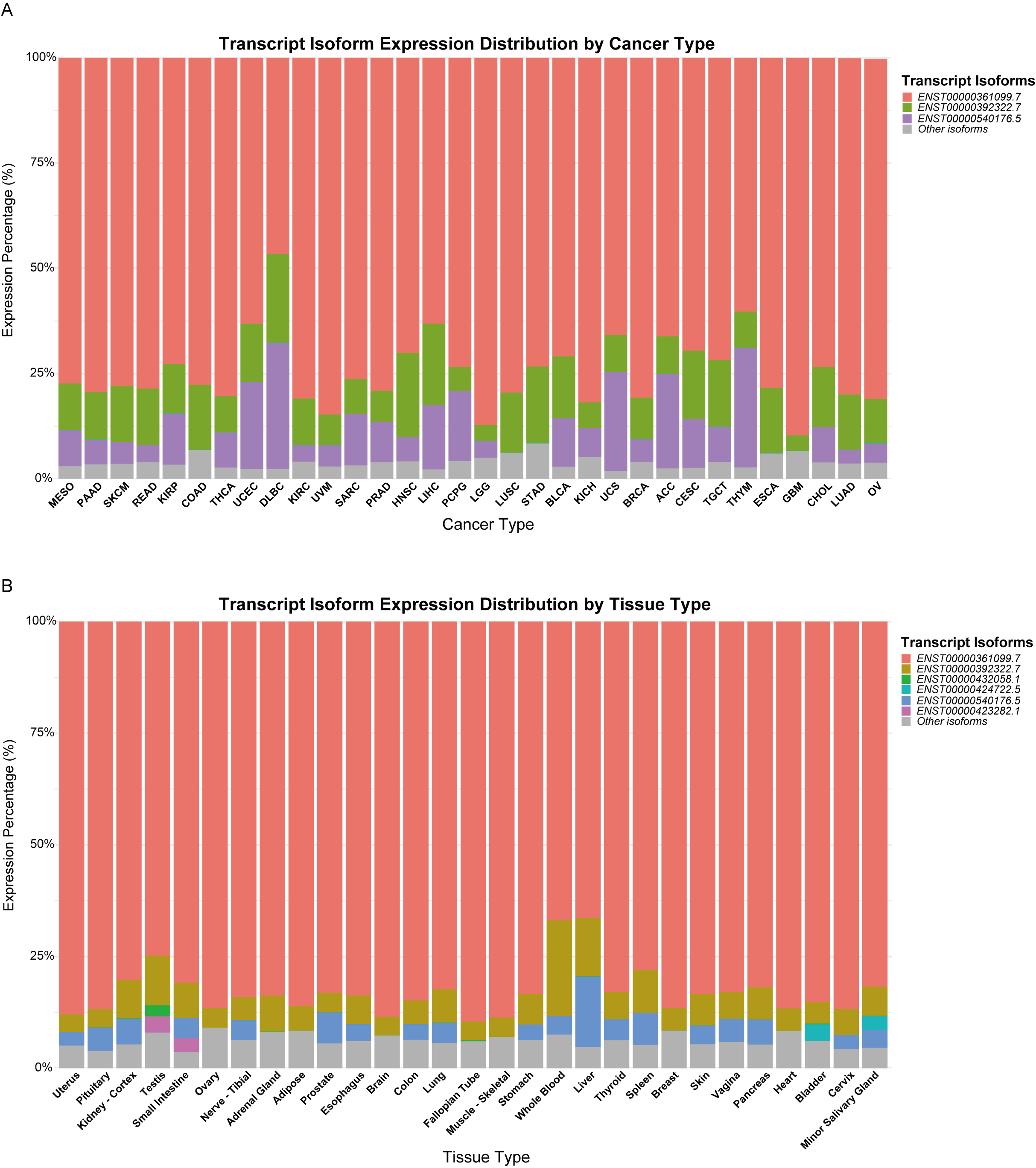


**Supplemental Figure 1.** Expression of *STAT1* transcripts in normal and cancer tissues. **(A-B)** Stacked bar plot illustrating the relative expression of the different transcript isoforms of *STAT1* among different cancer types (A) and normal tissues (B).


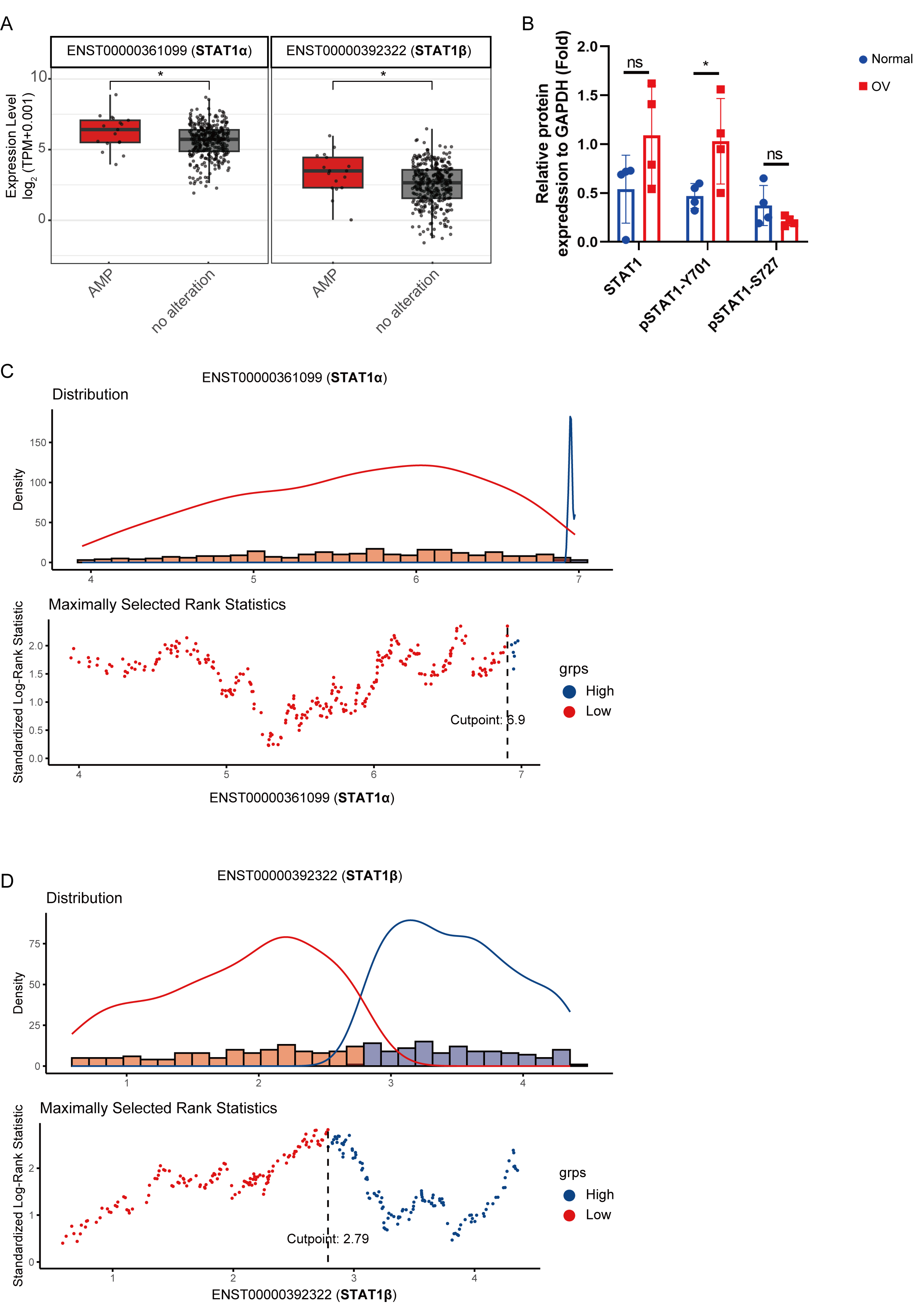


**Supplemental Figure2.** Association of *STAT1* transcript abundance with genomic amplification and optimal cutoff determination. **(A)** Boxplot showing the difference in expression levels of the two transcripts expressions between the ovarian cancer patients with *STAT1* amplification (red) and who without amplification (gray). Stats: Wilcoxon rank-sum test. *p < 0.05 **(B)** Quantitative analysis of STAT1 expression and phosphorylation patterns in ovarian cancer vs. normal ovarian tissues (n=4 independent experiments). Stats: Two-tail Student's t-test. *p < 0.05. (**C-D)** Optimal cutoff values were determined for ENST00000361099 (C) and ENST00000392322 (D).
